# Flexible bronchoscopy and cryoextraction for critical airway obstruction caused by an endobronchial angioleiomyoma

**DOI:** 10.1002/rcr2.415

**Published:** 2019-03-13

**Authors:** Sumit Chatterji, Efrat Ofek, Tiberiu Shulimzon

**Affiliations:** ^1^ Interventional Pulmonology Unit Pulmonary Institute, Sheba Academic Medical Center Tel Hashomer Israel; ^2^ Department of Pathology Sheba Academic Medical Center Tel Hashomer Israel

**Keywords:** Angioleiomyoma, bronchial, bronchoscopy, cryotherapy, obstruction

## Abstract

Angioleiomyomas are rare airway tumours with potential to cause central airway obstruction or haemoptysis. Methods described to manage them include surgical resection, or rigid bronchoscopy and thermal ablation techniques. We describe a case presenting with central airway obstruction, safely and effectively treated with cryoextraction of the tumour using flexible bronchoscopy.

## Clinical Image

A 63‐year‐old male was hospitalized with severe progressive dyspnoea.

He was obese (body mass index 36 kg/m^2^), an ex‐smoker of 20 pack‐years, with a medical history of diffuse large B‐cell lymphoma treated with chemotherapy and in remission for three years.

**Figure 1:**
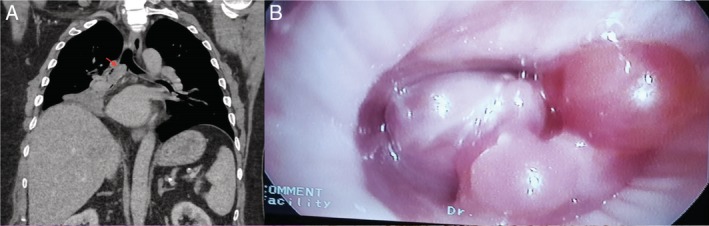
(A) Coronal reconstruction of computed tomography (CT) chest showing the bifurcation of trachea into right and left bronchial tree. Red arrow shows the endoluminal obstruction starting at the proximal right main bronchus and extending distally with lower lobe atelectasis. (B) Bronchoscopic image of multiple pink, smooth pedunculated lesions occluding the right main bronchus.

**Figure 2:**
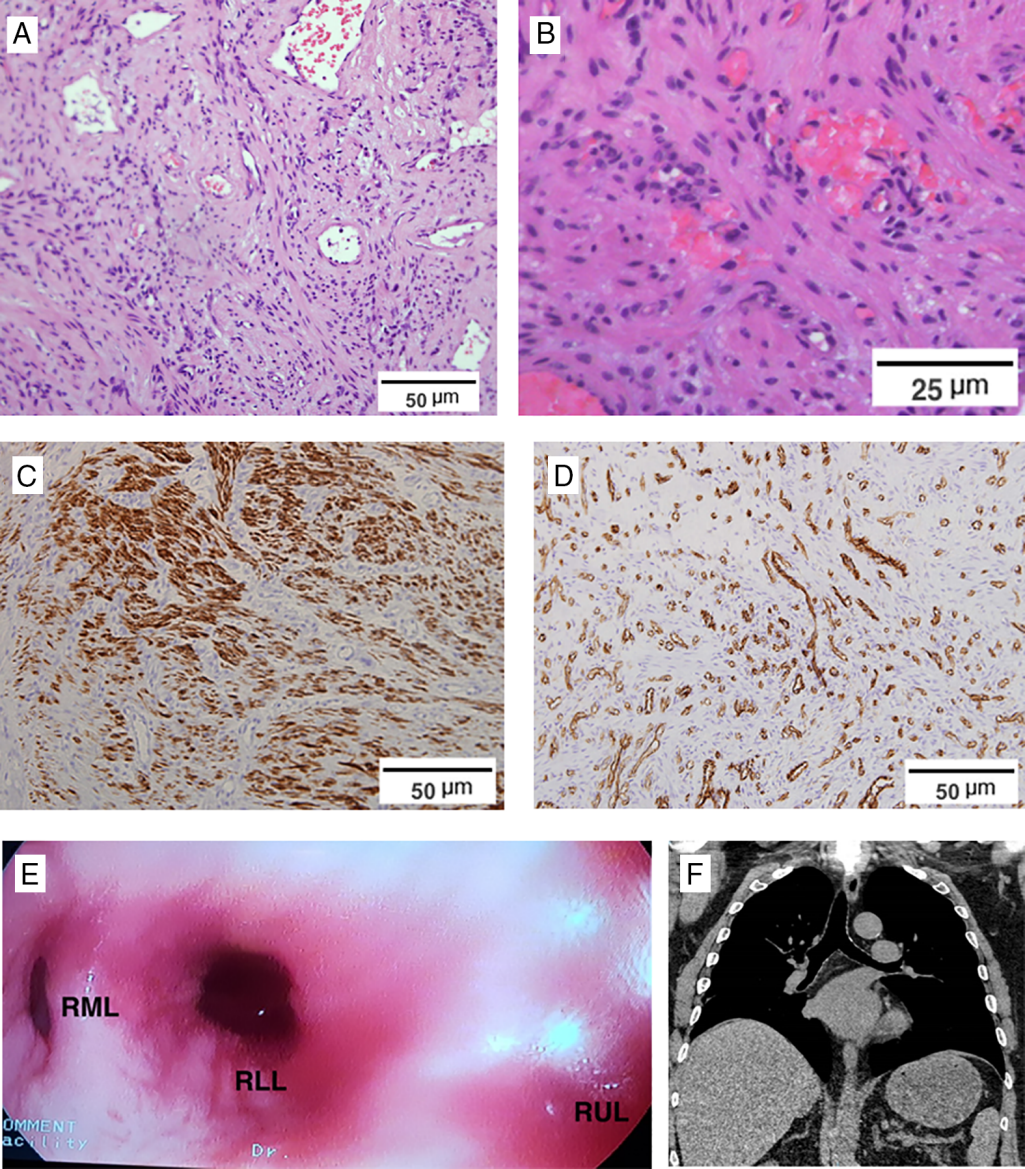
(A, B) Haematoxylin and eosin stains of the lesions at different magnifications showing submucosal tumour composed of bland‐looking, monomorphic spindled cells admixed with small and medium‐sized blood vessels. (C) Desmin immunostain highlighting muscle fibres and (D) CD31 immunostain for demonstrating endothelium. (E) Post‐cryoextraction appearance of the fully patent right bronchial tree (RLL, right lower lobe; RML, right middle lobe; RUL, right upper lobe just proximal to the image). (F) Post‐procedure coronal reconstruction of CT chest showing a clear right bronchial tree and no residual lower lobe atelectasis.

Chest computed tomography revealed an obstructing lesion in the right main bronchus with post‐obstructive pulmonary infiltrates (Fig. 1A). Diagnostic flexible bronchoscopy confirmed multiple smooth, pink polypoid masses occluding the right main bronchus extending into the bronchus intermedius (Fig. 1B). Forceps biopsy revealed an angioleiomyoma (Fig. 2A–D).

After discussion with thoracic surgery, considering factors including the proximity of disease to the main carina and extent of involvement of the right main bronchus, a decision was made to proceed initially with endobronchial debridement by interventional pulmonologists. Due to the possible risk of bleeding during the procedure, flexible bronchoscopy was performed in the operating room, under general anaesthesia. The patient was intubated with a size 8 cuffed endotracheal tube and there was immediate access to endobronchial balloon blockers and Neodymium:Yttrium Aluminium Garnet (Nd:YAG) laser for photocoagulation or thermal ablation. Cryoextraction using a 2.4‐mm diameter cryoprobe (Erbe GmbH, Germany) was performed with piecemeal removal of the multiple lesions. Complete airway clearance was successfully achieved with minimal bleeding (Fig. 2E,F).

Airway angioleiomyoma is a rare benign tumour arising from the tracheobronchial muscular fibres or blood vessels 1, 2. It has a prevalence of 2% of all airway tumours, and a slight male‐predominance 3.

Histologically, well‐differentiated smooth muscle cells are arranged around multiple vascular channels, with solid, venous, and cavernous variants. Immunohistochemistry for actin is typically positive, with desmin expression increased in the solid variant (75.6%) 4. Where primary surgery is not indicated, rigid bronchoscopy with thermal ablation techniques have been employed due to concerns about bleeding. However, flexible bronchoscopy and cryoextraction via endotracheal tube can be a safe and effective alternative when performed by interventional pulmonologists, as long as necessary equipment and expertise is available to deal with the possible bleeding complications.

### Disclosure Statement

Appropriate written informed consent was obtained for publication of this manuscript and accompanying images.
